# Self-medication practices and associated factors among households at Gondar town, Northwest Ethiopia: a cross-sectional study

**DOI:** 10.1186/s13104-019-4195-2

**Published:** 2019-03-19

**Authors:** Ebabu Jember, Amsalu Feleke, Ayal Debie, Geta Asrade

**Affiliations:** 1 Department of Pharmacy, College of Medical and Business, Addis Ababa, Ethiopia; 2 Department of Health Systems and Policy, Institute of Public Health, College of Medicine and Health Sciences, University of Gondar, P.O.Box 196, Gondar, Ethiopia

**Keywords:** Self-medication practices, Households, Factors, Gondar, Ethiopia

## Abstract

**Objective:**

Self-medication practice is the selection and use of medicines by individuals or a member of the individual’s family without physician’s order to treat self-recognized or self-diagnosed conditions. It is highly prone to inappropriate use and wastage of resources, increase drug resistance pathogens and adverse reactions. Therefore, this study aimed to assess self-medication practices and associated factors among households at Gondar town, Northwest Ethiopia.

**Results:**

This community based cross-sectional study was conducted among households at Gondar town from March to June, 2018. The overall prevalence of self-medication practices among households at Gondar town were 50.2%. The odds of self-medication practices among unmarried participants (AOR = 3.12; 95% CI 2.35, 5.34), influenced by peer (AOR = 3.58; 95% CI 2.89, 7.28), poor perceived quality of health care services (AOR = 4.67; 95% CI 2.56, 7.96) and access to pharmacy (AOR = 2.32; 95% CI 1.65, 6.76) were higher compared with their counterparts. In the contrary, the lesser odd was observed among knowledgeable participants about medications (AOR = 0.27; 95% CI 0.16, 0.39) compared with non-knowledgeable. Therefore, improving perception of participants about quality of services, conducting awareness creation and managing negative effects of peer may reduce self-medication practices.

**Electronic supplementary material:**

The online version of this article (10.1186/s13104-019-4195-2) contains supplementary material, which is available to authorized users.

## Introduction

Self-medication (SM) is the selection and use of medicines by individuals or a member of the individual’s family without physician’s order to treat self-recognized or self-diagnosed conditions [1]. It can help to treat minor ailments that do not require medical consultation and hence reduce the pressure on medical services, particularly in the deprived countries with inadequate health care resources. In a number of developing countries, many drugs are dispensed over the counter without medical direction [2].

Self-medication practices (SMP) is highly prone to inappropriate use and has its own drawbacks resulting wastage of resources, increase drug resistance pathogens and adverse reactions [3, 4]. It can also lead to incorrect self-diagnosis, delays in seeking appropriate care, dangerous drug interactions, risk of dependence, drug abuse, incorrect dosage and choice of medication [5, 6]. A systematic review reports indicated that prevalence of SMP was high globally and varies from 32.5 to 81.5% [7–11] and the average prevalence of SMPs in Ethiopia was also 36.8% [12].

Nowadays, drug resistance is becoming a worldwide problem, mainly in developing countries as a result of the availability and the use of antibiotics without prescription [13–15]. Accordingly, new forms of resistant pathogens can spread between continents with ease and this considered as “nightmare bacteria” that “pose a catastrophic threat” to people in every country in the world [16].

In many developing countries, antibiotics and potentially habit-forming medicines are easily available in every pharmacy sells without prescription. This together with poor awareness and lack of a good primary health care system coupled with cost issues cause the general public to buy drugs from private health institutions and shops without prescription [17].

In spite of this, there is limited literatures and no specific measures designed to address the problem in the country. Therefore, this study aimed to assess self-medication practices and associated factors among households at Gondar town, Northwest Ethiopia.

## Main text

### Methods

#### Study design and settings

A community based cross-sectional study was conducted among households at Gondar town from February to May, 2018. Gondar town is found in the northwest part of Ethiopia and it is 180, and 748 km far from Bahir Dar (capital city of Amhara region) and Addis Ababa, respectively. Based on the 2007 Central Statistical Agency (CSA) report of Ethiopia, the town had a total population of 400,000. From these, 198,120 (men) and 201,880 (women). The town has also 23 kebeles and 48 health institutions; these are: 1 comprehensive referral hospital, 8 health centers, 1 private general hospital, 15 speciality clinics, 15 medium clinics and 8 primary clinics.

#### Population and sampling procedure

All households in Gondar town were the source population, where as households in the selected kebeles of Gondar town were the study population. The sample size was determined using single population proportion formula with an assumption of the prevalence of SMP in Northwest Ethiopia (27.5%) [18], 95% Confidence Level (CL), 5% margin of error, 5% non-response rate and 2 design effect. The final sample size was 642. After sample size determination, five kebeles has been selected by using lottery method. Then the total sample size, 642, was proportionally allocated among the five selected kebeles based on the size of the households. The head of the household was our study participants. Accordingly, the interval was determined through dividing the total households to the total sample sizes and the first household was selected through lottery method among households within the first range of interval. Finally, systematic sampling technique was used to select participants.

#### Data collection tools and procedures

Interviewer administered structured questionnaire was used for data collection. The questionnaire was developed from reviewing different literatures [19–24]. The questionnaire was first prepared in English, then translated to Amharic and finally back to English by language expertise in order to maintain its consistency. Pre-test was also conducted among 32 households at Bahir Dar town and any ambiguity was modified based on pre-test findings. A total of 6 diploma graduated pharmacy technicians were recruited for data collectors and 3 Bachelor degree graduated pharmacists were recruited for supervisors. A-day training was given for data collectors and supervisors about the basic techniques of the data collection procedures.

#### Measurements

Self-medication practice, the outcome variable, was assessed by the selection and use of medicines/drugs by individuals or a member of the individual’s family with out physician’s order to treat self-recognized or self-diagnosed conditions in the past 6 months [25]. Accordingly, knowledge of participants about medications was measured by using eight item questions and each item contains (0 = no and 1 = yes) alternatives, and respondents who scored ≥ 50% of the total knowledge measuring scores were considered as knowledgeable.

#### Data management and analysis

Data were entered and analyzed using Epi-Info version 7.1 and SPSS version 20, respectively. Descriptive statistics such as frequencies and percentages were presented using tables, graphs, and texts. Variables with p-value < 0.2 during bivariable analysis were fitted to multiple logistic regression analysis. Finally, Adjusted Odds Ratio (AOR) with 95% CI and p-value less than 0.05 were used to determine variables significantly associated with SMPs.

### Results

#### Socio-demographic and economic characteristics

A total of 632 participants were participated with a response rate of 98.4%. About 60.8% of participants were females. 71.4% of respondents aged 30–45 years and the mean age of respondents was 36 ± 11 SD years. More than two-thirds (68.5%) of participants were Orthodox Christians and about 57% of the respondents were married. About 30% of participants had less than ETB 1000 (USD 37.7) monthly household income and attended their secondary school. Nearly one-fifth (18.5%) of the participants were government employee and more than 20% (21.4%) of participants had five or more family members (Table 1).

**Table 1:** Socio-demographic and economic characteristics of the respondents at Gondar town, Northwest Ethiopia, 2018

Variables	Frequency	Percent (%)
*Sex of participants*		
Male	248	39.2
Female	384	60.8
*Age in years*		
< 30	70	11.1
30–45	451	71.4
≥ 45	111	17.6
*Religion*		
Orthodox	433	68.5
Muslim	181	28.6
Protestant	18	2.9
*Marital status*		
Single	267	42.2
Married	365	57.8
*Educational status*		
Unable to read and write	113	17.9
Primary school	166	26.3
Secondary school	193	30.5
College diploma and above	160	25.3
*Monthly household income*		
ETB < 1000 (< USD 37.7)	189	29.9
ETB ≥ 1000 (≥ USD 37.7)	443	70.1
*Family size*		
< 5	497	78.6
≥ 5	135	21.4
*Occupation*		
Govt employee	117	18.5
Merchant	299	47.3
Daily laborer	103	16.3
Others	113	17.9

#### Self-medication practices

The prevalence of SMP among households in the past 6 months at Gondar was 50.2%. more than 60% (60.9%) of the household had history of SMPs within their family members in the town, but they didn’t remember the name of the drug. About 13.2% of the participants encounter some drug adverse reactions and more than 40% (43.2%) had checked the expiry date of the drug before they use. Similarly, almost half (49.5%) were concerned that increasing the dose of the drug can be hazardous for health. About 46% of respondents had access to pharmacy and more than half (56%) of participants were influenced by their peer/s (Table 2).

**Table 2:** Self-medication practices at households of Gondar town, northwest Ethiopia, 2018

Variables	Frequency	Percent (%)
*Self-medication practices*		
No	315	49.8
Yes	317	50.2
*Did you remember the name of the drug?*		
No	193	60.9
Yes	124	39.1
*Did you encounter any adverse drug reaction during self-medication practices?*		
No	275	86.8
Yes	42	13.2
*Did you check the expiry date of the drugs before use?*		
No	180	56.8
Yes	137	43.2
*Did you have any concern that increasing drug dose can be hazardous for health?*		
No	160	50.5
Yes	157	49.5
*Did you ever check the instructions/leaflets within the drugs package during self-treatment?*		
No	232	36.7
Yes	85	13.4
*Access to pharmacy*		
No	341	54.0
Yes	291	46.0
*Peer pressure*		
No	278	44.0
Yes	354	56.0

#### Source of information about SMPs

More than one-third (35.3%) of the sources of information for their households about medication practices were pharmacy professionals and previous experience was also the sources of information for around 30% of the participants. On the other hand, one-third (67.8%) of the respondents had bought the drugs for SMPs were private drug stores followed by 11.7% private clinic. About 45.4% participants were knowledgeable about medications, one-fifth (22.2%) of respondents had good perceived quality of governmental health care services and 42.4% perceived the costs of SMs was cheap (Additional file 1: Table S1).

#### Reasons and common symptoms for SMPs

Participants reported that the reasons for their households SMPs were severity of illnesses (44.8%), emergency cases (35.3%), reducing medical cost (17%), lack of trust by the prescribers (4.1%) and for saving time (5.1%) (Additional file 2: Fig S1).

The common symptoms/illnesses that prompted SMs among participants who had practiced self-medications were headache (63.1%), RTIs (cough, cold/flu) (30.9%), fever (18.9%), GIT infections (diarrhea, vomiting) (38.9%) and dysmenorrhea (7.6%) (Additional file 3: Fig S2).

#### Factors associated with SMPs

Binary logistic regression model was used for analysis. AOR with 95% CI and p-value less than 0.05 were used to determine variables associated with SMPs. The odds of SMPs was higher among unmarried (AOR = 3.12; 95% CI 2.35, 5.34), influenced by peer pressure (AOR = 3.58; 95% CI 2.89, 7.28), access to pharmacy (AOR = 2.32; 95% CI 1.65, 6.76) and poor perceived quality of governmental health care services (AOR = 4.67; 95% CI 2.56, 7.96) compared with that of their counterparts. On the other hand, lesser odds of SMP was observed among knowledgeable participants about medications (AOR = 0.27; 95% CI 0.16, 0.39) than that of non-knowledgeable (Table 3).

**Table 3:** Multiple logistic regression analysis for factors associated with SMPs at Gondar town, Northwest Ethiopia, 2018

Variables	Self-medication practices		COR (95% CI)	AOR (95% CI)	p-value
	Yes	No			
*Sex of participants*					
Male	127	121	1	1	
Female	190	194	0.93 (0.68, 1.28)	0.75 (0.53, 1.51)	0.51
*Age in years*					
< 30	40	30	1	1	
30–45	210	241	0.65 (0.39, 1.09)	0.51 (0.25, 1.25)	0.18
≥ 45	67	44	1.14 (0.62, 2.10)	1.63 (0.38, 3.12)	0.46
*Marital status*					
Single	157	110	1.83 (1.33, 2.52)	3.12 (2.35, 5.34)*	0.01
Married	160	205	1	1	
*Peer pressure*					
No	82	196	1	1	
Yes	235	119	4.72 (3.36, 6.63)	3.58 (2.89, 7.28)*	0.001
*Monthly household income*					
< ETB 1000 (< USD 37.7)	107	82	1	1	
≥ ETB 1000 (≥ USD 37.7)	210	233	0.69 (0.49, 0.97)	0.52 (0.34, 1.17)	0.32
*Access to pharmacy*					
No	223	118	1	1	
Yes	199	92	4.09 (2.93, 5.70)	2.32 (1.65, 6.76)*	0.001
*Family size*					
< 5 members	255	242	1	1	
≥ 5 members	62	73	0.81 (0.55, 1.18)	0.62 (0.37, 1.39)	0.25
*Perceived quality of health care*					
Good	54	86	1	1	
Poor	263	229	1.83 (1.25, 2.68)	4.67 (2.56, 7.96)*	0. 001
*Perceived cost*					
Expensive	181	183	1	1	
Cheap	136	132	1.04 (0.76, 1.43)	2.17 (0.64, 5.84)	0.67
*Knowledge*					
Not knowledgeable	249	96	1	1	
Knowledgeable	68	219	0.12 (0.08, 0.17)	0.27 (0.16, 0.39)*	0.01

* Significant at *p*-value < 0.05

### Discussion

This study was designed to assess the prevalence of SMPs and associated factors among households at Gondar town, Northwest Ethiopia. In our survey, prevalence of SMPs in the past 6 months was 52.2%. The finding was higher than studies done at University of Gondar (38.5%) [19], Mekelle (43.24%) [20], Meket district (35.9%) [26], Nekemte (36.7%) [27], Northwest Ethiopia (27.5%) [18], Puducherry, India (11.9%) [28], Saudi Arabia (35.4%) [29] and Haman dan province, Iran (35.4%) [30]. However, it was lower than studies done in Harar (57.8%) [31], Addis Ababa (72.8%) [21], Sri Lankan (60.8%) [32], Pune (87.5%) [33], Belgrade, Serbia (79.9%) [34], Oman University (94%) [25], Pokhara, Nepal (59%) [22], Italy (69.2%) [35], Barabanki (69.6%) [36], Delhi, India (92.8%) [37], Turkey (63.4%) [38], private University Malaysia (77.6%) [39], Mbeya, Tanzania (55.7%) [40], Gulbarga Karnataka, India (88.2%) [41], Karachi, Pakistan (76%) [24] and Egypt (62.9%) [42]. This variation may be due to the differences in social determinants of health, beliefs, culture of the population, and variation on recall periods used in each study.

Our finding indicated that the odds of SMPs among unmarried participants were 3.12 times higher than the odds of married participants. This finding was consistent with the studies conducted in Meket district, Ethiopia [27] and Udupi Taluk, southern India [43]. This might be so because unmarried participants could be influenced by peer pressure. The odds SMPs among households who were influenced by peer pressure were 3.58 times higher than that of the respondents who didn’t influence by their peers. This study was in line with other studies done in Meket [27], China [44] and Uganda [45]. The possible explanation might be due to the source of information for peers/friends might be the same. Similarly, they may have similar life experience and can also share their own previous practices between them. This could also work in the case of self-medications.

Our study report also indicated that the odds of SMPs among knowledgeable participants were lesser by 73% compared with their counterparts. This might be due to knowledgeable community members may fear the bad adverse reactions self-medications. Accordingly, the odds of SMPs among participants who had poor perceived quality of health care services in the governmental health care facilities were 4.67 times higher than respondents who had good perceived quality of health care. The possible explanation might be so because participants who had poor perceived quality health care services may decide that the role of physician’s diagnosis is not significant for treating their health problems.

In this study, the odds of SMPs among respondents who had an access to pharmacy were 2.32 times higher compared with the participants who had no access. This finding was supported by studies conducted in Meket, Ethiopia [27], China [44], southern India [43] and Nigeria [46]. The possible justification might be due to the inability of the participants to afford health care fees and lack of time to consult health care professionals. Therefore, improving perception of participants about the quality of health care services, awareness creation and managing peer pressure may reduce self-medication practices.

## Limitations

- This study was conducted in urban setting which didn’t show the practices of the rural area.
- The study was assessing only about modern medicines which did not include practices of traditional medications that might be under estimate our findings in the study area.
- The study might also prone to recall bias as a result of self-reporting of the participants for their last 6 months experiences.

## Additional files

**Additional file 1: Table S1.** Sources of information and drugs for SMPs among households at Gondar town, Northwest Ethiopia, 2018.

**Additional file 2: Fig. S1.** Reasons for self-medication practices among households at Gondar town, Northwest Ethiopia, 2018 (n = 317).

**Additional file 3: Fig. S2.** Common symptoms/illnesses that prompted self-medications among households at Gondar town, Northwest Ethiopia, 2018 (n = 317).

## Abbreviations

AOR: Adjusted Odds Ratio
CI: Confidence Interval
COR: Crude Odds Ratio
GIT: Gastro-Intestinal Tract
RTIs: Respiratory Tract Infections
SM: Self-Medications
SMP: Self-Medication Practice
WHO: World Health Organization
