# Supplementary material for: Self-medication practices and associated factors among households at Gondar town, Northwest Ethiopia: a cross-sectional study
Source: BMC Res Notes. 2019 Mar 19;12:153. doi: 10.1186/s13104-019-4195-2 (PMC6425615; doi:10.1186/s13104-019-4195-2)
Supplement: Supplementary file 1 — Additional file 1: Table S1. Sources of information and drugs for SMPs among households at Gondar town, Northwest Ethiopia, 2018. [file 13104_2019_4195_MOESM1_ESM.docx]

Table S1: Sources of information and drugs for SMPs among households at Gondar town,2018

| Variables | Frequency | Percent (%) |
| --- | --- | --- |
| **From whom did you get the information? (n= 317)** |  |  |
| Pharmacy professionals | 112 | 35.3 |
| Previous prescription | 28 | 8.8 |
| Previous experience | 96 | 30.3 |
| Others | 93 | 29.4 |
| **Where did you get the drugs? (n=317)** |  |  |
| Government hospital | 22 | 6.9 |
| Private hospital | 32 | 10.1 |
| Health center | 16 | 5.1 |
| Private clinic | 37 | 11.7 |
| Private drug store | 215 | 67.8 |
| Others | 14 | 4.4 |
| **Knowledge about medications(n=632)** |  |  |
| Not knowledgeable | 345 | 54.6 |
| Knowledgeable | 287 | 45.4 |
| **Perceived quality health care services(n=632)** |  |  |
| Good | 140 | 22.2 |
| Poor | 492 | 77.8 |
| **Perceived cost of self-medication(n=632)** |  |  |
| Expensive | 364 | 57.6 |
| Cheap | 268 | 42.4 |
