# Supplementary figures and images for: Self-medication practices and associated factors among households at Gondar town, Northwest Ethiopia: a cross-sectional study
Source: BMC Res Notes. 2019 Mar 19;12:153. doi: 10.1186/s13104-019-4195-2 (PMC6425615; doi:10.1186/s13104-019-4195-2)

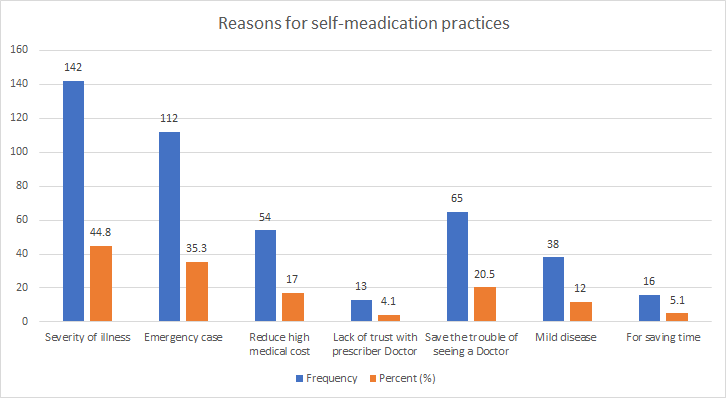

Supplement: Supplementary file 2 — Additional file 2: Fig. S1. Reasons for self-medication practices among households at Gondar town, Northwest Ethiopia, 2018 (n = 317). [file 13104_2019_4195_MOESM2_ESM.tif]

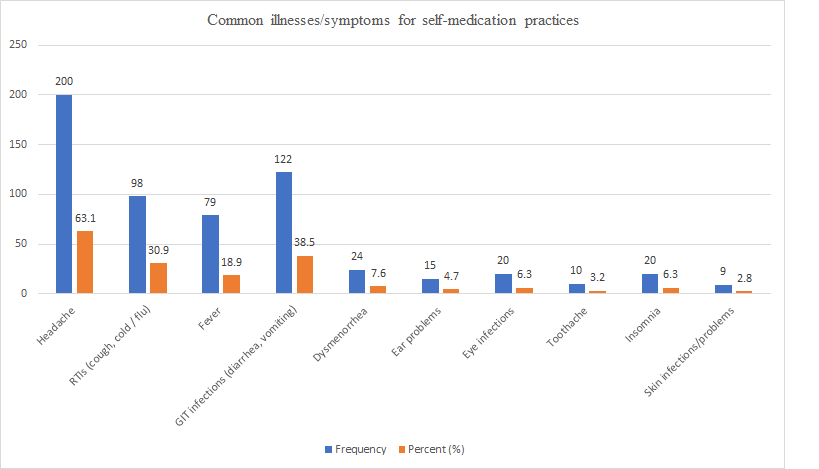

Supplement: Supplementary file 3 — Additional file 3: Fig. S2. Common symptoms/illnesses that prompted self-medications among households at Gondar town, Northwest Ethiopia, 2018 (n = 317). [file 13104_2019_4195_MOESM3_ESM.tif]
